# The human platelet: strong transcriptome correlations among individuals associate weakly with the platelet proteome

**DOI:** 10.1186/1745-6150-9-3

**Published:** 2014-02-14

**Authors:** Eric R Londin, Eleftheria Hatzimichael, Phillipe Loher, Leonard Edelstein, Chad Shaw, Kathleen Delgrosso, Paolo Fortina, Paul F Bray, Steven E McKenzie, Isidore Rigoutsos

**Affiliations:** 1 Computational Medicine Center, Thomas Jefferson University, Philadelphia, PA 19107, USA; 2 Cardeza Foundation for Hematologic Research, Division of Hematology, Department of Medicine, Thomas Jefferson University, Philadelphia, PA 19107, USA; 3 Department of Molecular and Human Genetics, Baylor College of Medicine, Houston, TX 77030, USA; 4 Cancer Genomics Laboratory, Kimmel Cancer Center, Department of Cancer Biology, Thomas Jefferson University, Philadelphia, PA 19107, USA; 5 Department of Molecular Medicine, Universita’ La Sapienza, Rome, Italy

## Abstract

**Background:**

For the anucleate platelet it has been unclear how well platelet transcriptomes correlate among different donors or across different RNA profiling platforms, and what the transcriptomes’ relationship is with the platelet proteome. We profiled the platelet transcriptome of 10 healthy young males (5 white and 5 black) with no notable clinical history using RNA sequencing and by Affymetrix microarray.

**Results:**

We found that the abundance of platelet mRNA transcripts was highly correlated across the 10 individuals, independently of race and of the employed technology. Our RNA-seq data showed that these high inter-individual correlations extend beyond mRNAs to several categories of non-coding RNAs. Pseudogenes represented a notable exception by exhibiting a difference in expression by race. Comparison of our mRNA signatures to a publicly available quantitative platelet proteome showed that most (87.5%) identified platelet proteins had a detectable corresponding mRNA. However, a high number of mRNAs that were present in the transcriptomes of all 10 individuals had *no* representation in the proteome. Spearman correlations of the relative abundances for those genes represented by both an mRNA and a protein showed a weak (~0.3) connection. Further analysis of the overlapping and non-overlapping platelet mRNAs and proteins identified gene groups corresponding to distinct cellular processes.

**Conclusions:**

The results of our analyses provide novel insights for platelet biology, show only a weak connection between the platelet transcriptome and proteome, and indicate that it is feasible to assemble a platelet mRNA-ome that can serve as a reference for future platelet transcriptomic studies of human health and disease.

**Reviewed by:**

This article was reviewed by Dr Mikhail Dozmorov (nominated by Dr Yuri Gusev), Dr Neil Smalheiser and Dr Eugene Koonin.

## Background

Platelets circulate in the blood and are involved in central physiological processes such as hemostasis, wound healing and host defense. Following their release into the blood stream from the megakaryocytes in the bone marrow, platelets from healthy individuals have an average lifespan of seven to ten days. Through their interactions with leukocytes and endothelial cells, platelets play an important role in angiogenesis, the storage of bioactive molecules, and the production and secretion of pro- and anti-inflammatory molecules [1]. Abnormal platelet number and function cause or contribute to a variety of diseases including hemorrhagic diseases, pathologic thrombosis, atherosclerosis, and cancer metastases. Despite many advances in elucidating platelet biology, gaps in our understanding of the molecular mechanisms underlying platelet function persist.

Although much of the platelet transcriptome is inherited from the megakaryocyte from which they derive [2], platelets can actively splice and post-transcriptionally regulate mRNAs [3] and translate proteins [4-8]. Given the presence of proteins and the absence of active transcription, the concordance or lack thereof between the platelet’s transcriptome and proteome has been a topic of long-standing research focus. Based on a limited number of platelet proteomic [9] and transcriptomic [6,10] studies, a correlation between the two has yielded contradictory results and the issue remains controversial [11,12]. Having a more complete understanding of the relationship between the two will aid in our understanding of platelet biology.

Next-generation sequencing (NGS) of platelets has enabled unprecedented characterization and quantification of the platelet transcriptome and revealed an unexpectedly diverse repertoire of mRNAs, microRNAs (miRNAs), other non-coding RNAs (ncRNAs) [6,10]. These initial transcriptome profiles were generally in agreement with earlier microarray-based efforts [13,14]. We are aware of RNA-seq experiments on only five platelet samples: a pool of two healthy donors [6] and four healthy white males [10]. We now report the largest series to date using both RNA-seq and microarray technologies to characterize the platelet transcriptome. We also report on the use of the obtained RNA-omes in gauging the extent of *inter*-individual correlations. Additionally, we examined the existence of expression links across the two racial groups. Lastly, we carried out and report on comparative analyses of the various subsets of mRNAs that are concordant and discordant with the recently reported quantitative proteome [15].

## Methods

### Samples, DNA, and RNA preparation

The study was approved by the Institutional Review Board of Thomas Jefferson University and informed consent was obtained for all participants. Peripheral blood samples were collected from 10 healthy males with no previous history of thrombosis or history of bleeding who were taking no anti-platelet medications (see Additional file 1 for demographics information). DNA was extracted from the buffy coat preparations of the subjects using the Gentra Puregene Blood Kit (Qiagen, Netherlands). DNA was hybridized to the HumanOmni5 array (Illumina Inc, San Diego, CA) at the laboratory for Translational Genomics at the Baylor College of Medicine. RNA was extracted from highly purified leukocyte-depleted platelets (LDPs) using magnetic beads (Miltenyi Biotec) against CD45 for leukocyte depletion as previously described [16]. Each of 10 individuals was genotyped with the help of 2 million genome-wide markers [17]. In addition, each individual self-identified himself racially. Our analyses show complete agreement between each subject’s self-identified race and genotype Additional file 2).

### RNA sequencing

We have shown that ribosomal RNA (rRNA) depletion from platelet RNA preparations impacts adversely and non-uniformly on the relative abundance of transcripts [10]. Consequently, we sequenced *total* RNA to avoid skewing of the estimates of relative abundance of the various molecular categories. Total RNA sequence library construction, emulsion PCR, and sequencing runs were performed following the Applied Biosystems/Life Technologies protocols, and sequencing was performed on the SOLiD 5500xl platform. The total RNA was size selected and for each sample, RNA libraries between 150 and 500 nucleotides (nts) were generated (referred to throughout as “long RNA” to distinguish from small RNA libraries that query microRNA, for example) and 50 nt reads were sequenced using a single-end approach. No multiplexing was used.

### Read mapping

Sequence reads were mapped onto the human genome assembly hg19 using the Short Read Mapping Package (SHRiMP) [18]. Prior to mapping, quality-based trimming was performed on the sequence reads using the *cutadapt* tool [19]. During mapping we allowed mismatches (replacements) that comprised not more than 4% of a given read’s length; we did not permit any insertions or deletions. This stringency is aimed at minimizing the instances of falsely mapped reads, i.e. of reads mapping to regions to which they do not belong. Also, mapped reads *shorter* than 16 nts were discarded and not considered further. For our analyses, we only used reads that mapped uniquely to the genome under these conditions. Those sequence reads that could not be mapped to the genome at all were also excluded from further analysis.

### Annotation of mapped reads

The genomic regions to which the sequenced reads mapped were analyzed using genomic annotations obtained from several public repositories. For protein-coding genes, pseudogenes, and lncRNAs, we used the annotations contained in the ENSEMBL database (http://www.ensembl.org/). We also considered the 14 classes of repeat elements and ncRNAs used by RepeatMasker [20]: DNA and RNA repeats, long interspersed nuclear elements (LINEs), short interspersed nuclear elements (SINEs), long tandem repeats (LTRs), RCs, Simple Repeats, ribosomal RNAs (rRNAs), Satellites, small cytoplasmic RNAs (scRNAs), small nuclear RNAs (snRNAs), signal recognition particle RNAs (srpRNAs), transfer RNAs (tRNAs), and the class “Others or Unknown”. The genomic coordinates for these genomic features were extracted from the Tables of the UCSC human genome browser (http://genome.ucsc.edu). For our analysis, we stringently defined ‘*purely intronic* regions’ to be “those segments of known unspliced pre-mRNA that remain after removing all known genomic features that are sense to the pre-mRNA such as exons, miRNAs, repeat elements, etc.” Analogously, we stringently defined ‘*unannotated* intergenic regions’ to be “those segments of the genome that remain after removing all protein coding loci as well as all other already-characterized genomic features”.

### Gene expression

For RNA-seq data, gene expression levels were approximated using the RPKM (reads per kilobase per million mapped reads) measure [21] and further normalized using the *β*-actin transcript (ENSEMBL identifier ENST00000331789). As we showed previously the resulting expression estimates correlate very well with qRT-PCR across a very wide dynamic range [10]. We employed very stringent abundance thresholds and only considered transcripts whose expression was ≥ 1/10,000 of *β*-actin’s expression (~13 PCR cycles): using our approach, our least abundant transcripts across the 10 datasets have RPKM values that are *higher* than the 0.3-0.5 RPKM thresholds used in similar studies. Our analyses are therefore more stringent in that they are confined to sets of mRNAs that are more abundant than what is typically considered. For microarray data, gene expression levels are estimated using the Affymetrix GeneChip and associated manufacturer software. Microarray data were further normalized using robust multichip averaging (RMA), background-corrected, quantile-normalized and log2-transformed.

### Determination of feature enrichment

For genomic regions belonging to a given category (i.e. ‘exon’, ‘rRNA’, ‘miRNA’, etc.) we calculated enrichment as the ratio of bases in the category that are covered by mapped reads (“observed”) over the bases that would have been covered by the mapped reads if this were a random process (“expected”). We calculated P-values by shuffling multiple times the genomic locations of the category under consideration and generating a distribution of the enrichments for the shuffled regions; a minimum of 1,000 reshufflings were performed in each case. Elements were considered enriched if they displayed an enrichment of at least a ±1.5 fold change and had a P-value < = 0.05.

### Platelet proteome

We used the quantitative proteome set that was reported recently [15]. We pre-processed the original set of ~4200 entries keeping only those that had a reported confidence estimate of 99% or 100%. We also removed duplicate entries keeping only the most abundant among the duplicates. We did not consider the more recent addition of 24 entries [12] in order to facilitate comparisons with other reports [11,12]. The resulting set contained 3544 unique UNIPROT identifiers. We used the recent quantitative proteome data together with data from two earlier reports [9,22] to facilitate identification of qualitative relationships.

### Statistical analyses

Pair-wise Pearson correlations were calculated using the normalized expression levels of the various features (e.g. mRNAs, pseudogenes, etc.) between two individuals. To compare the transcriptome vs. the proteome, a ranked Spearman correlation was used to compare the abundances of the overlapping expressed features.

### Gene ontology analyses

Gene ontology (GO) analyses were carried out using DAVID [23,24] (http://david.abcc.ncifcrf.gov/). For these analyses, we enforced very stringent settings for “ease” (Ease = 0.00001), P-value (≤ 0.00001), and “false discovery rate” (≤ 0.01) together with a minimum fold enrichment of 1.7.

### Principal components analysis (PCA)

We carried out PCA of the 2 million genome-wide genotype markers in the 10 subjects. To this end, we used the Eigenstrat software package [25] to compute the PCA transformation, excluding ethnicity information from the analysis.

### Data access

The RNA-seq and microarray data that we have generated for the 10 individuals are available through GEO ids SRP028846 and GSE50858 respectively. The mapped RNA-seq data can also be explored interactively at https://cm.jefferson.edu/platelets_2014/

## Results

### The Platelet Transcriptomes of 10 healthy donors by RNA-Seq

RNA-seq was performed on total RNA from highly purified platelets from 10 healthy male donors (see Additional files 1 and 2 for subject demographics). Across all 10 individuals, we generated nearly 1.6 billion sequence reads (long RNA-seq) with an average of 158 million reads per sample (Additional file 3). Approximately 41% of the reads that were sequenced mapped unambiguously to the human genome, a rate typical for whole genome RNA-seq. A combined total of ~650 million uniquely mapped sequence reads were used in subsequent analyses.

### Protein-coding mRNAs

**Figure 1:**
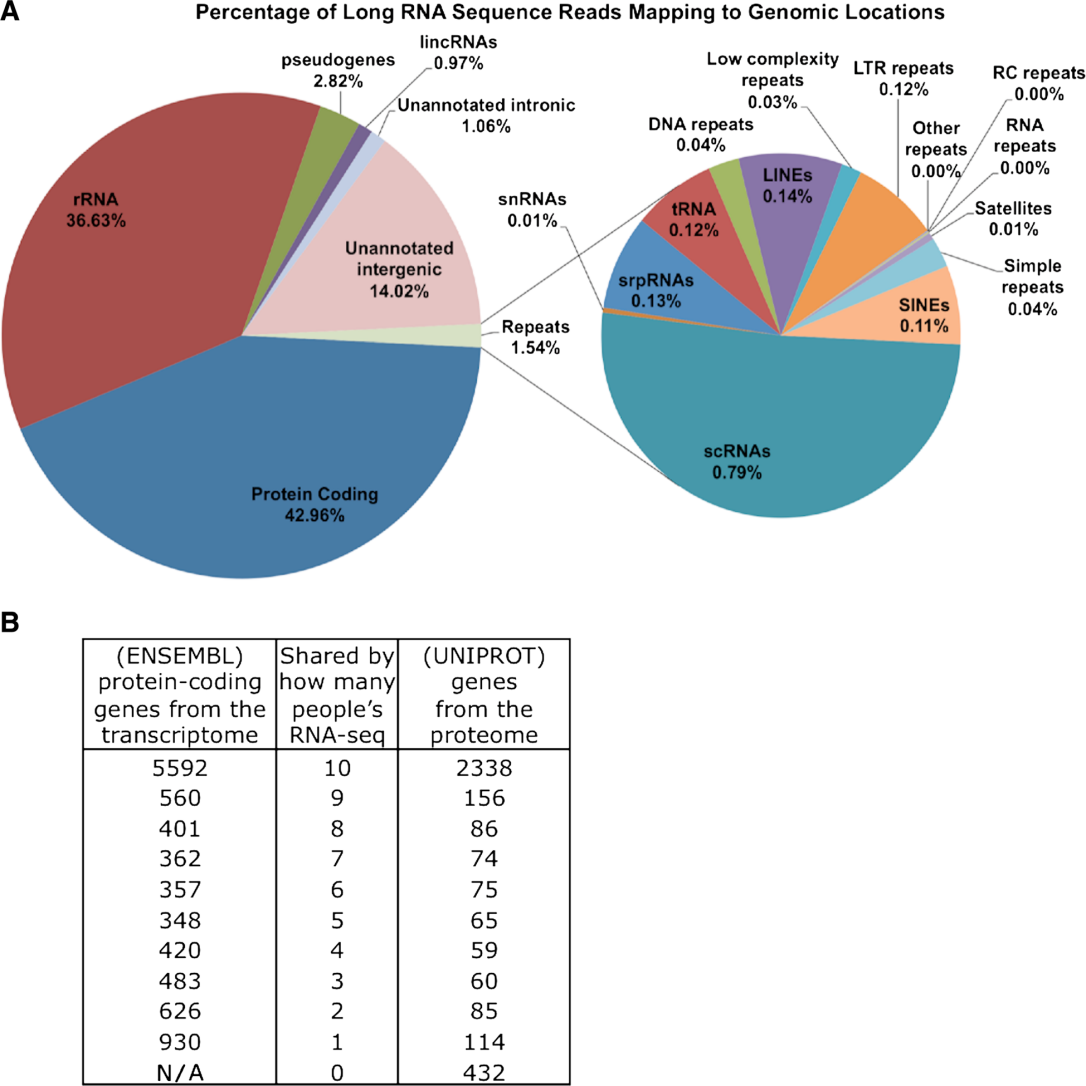
**Reads, genes and the genome. A)** Percentage of mapped reads across annotated genomic regions. Shown is the average percentage of uniquely mapped reads (long RNAs) that land on different genomic regions for the 10 samples. **B)** Table showing how many individuals share how many of the sequenced mRNAs (RNA-seq) and proteins (proteome reported in Burkhart et al [15]).

Of the uniquely mapped long RNA-seq reads ~43.0% are accounted for by genomic loci that correspond to the mRNAs of protein coding genes, ~36.6% map to rRNA, 14.0% to unannotated intergenic space, and the remaining ~6.0% to non-protein-coding loci (Figure 1A). For the stringent abundance thresholds that we use (≥ 1/10,000^th^ of β-actin – see Methods), we find that the transcripts from the 10 individuals represent a combined total of 10,079 distinct protein-coding genes, i.e. *more than half* of the known human protein coding genes. Of these genes, roughly 50% (5,592, Table 1, Additional file 4) of these genes are present in all 10 individuals (Figure 1B, and the data can also explored at https://cm.jefferson.edu/platelets_2014/). The number of shared expressed genes increases when considering smaller subsets of the 10 individuals. For example, ~7,000 of the 10,079 genes (~69%) are expressed in platelets from seven or more of the 10 individuals (Figure 1B). The entries in Figure 1B indicate that the number of mRNAs that are expressed by any two of the 10 individuals is high.

**Table 1:** Categories of platelet transcripts

**Category**	**Average number of expressed elements**	**Number of elements** ***in intersection*** **of all 10 samples**	**Number of elements in** ***union*** **of all 10 samples**
Protein coding genes	7,590	5,592	10,079
pseudogenes	1,275	706	2,356
lncRNAs	151	80	287
DNA repeats	1,986	223	3,833
LINE	5,012	591	8,545
Low complexity repeats	1,613	310	4,079
LTR repeats	2,725	508	3,676
Other repeats	24	9	16
RC repeats	8	2	14
RNA repeats	34	9	1,522
Simple repeats	2,846	410	9,980
rRNA repeats	213	135	268
Satellites	15	4	23
scRNA	447	272	574
SINE	10,319	911	13,782
snRNA	60	18	113
srpRNA	260	124	275
tRNA	123	47	253
Unknown repeats	32	6	56
(Purely) Intronic	28,636	4,323	161,826
(Unannotated) Intergenic	9,876	2,208	41,666

### Inter-individual correlations of mRNA transcripts

**Figure 2:**
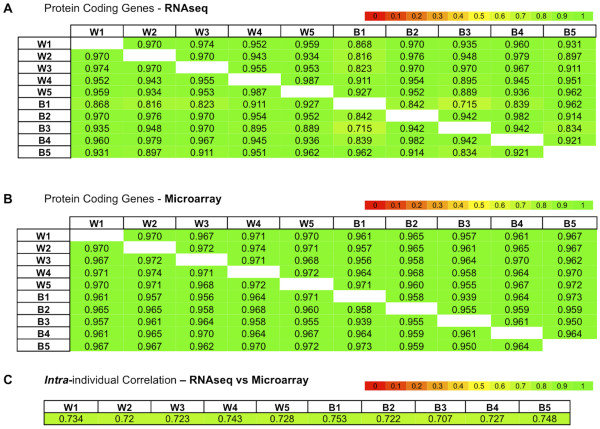
**Inter- and intra-individual correlations. A)** Heatmap of the inter-individual correlation of all mRNA transcripts (RNA-seq). **B)** Heatmap of the inter-individual correlation of all mRNA transcripts (microarray). **C)** Heatmap of the intra-individual correlation of all mRNA transcripts (RNA-seq vs microarray). The sample IDs are labeled with a W (White) or B (Black).

Next, we computed the inter-individual (pair-wise) Pearson correlations using the normalized RNA-seq expression of mRNA transcripts shared between any two individuals. We found the mRNA transcriptome profiles of the 10 individuals to be very highly correlated (Figure 2A). This result in conjunction with the results of Figure 1B indicates that for a large fraction of the captured mRNA profiles the *composition* and *relative abundance* of the corresponding mRNA transcripts are consistently similar across the 10 individuals. To ensure that the observed high correlations are not related to the employed technology, we also profiled the mRNAs of the same 10 individuals using a gene expression microarray. As in the case of RNA-seq, microarray profiling revealed high inter-individual Pearson correlation of the mRNA transcripts (Figure 2B). The very high correlations shown in Figures 2A and 2B did not materially change when we analyzed only the subset of genes for which a protein was identified by Burkhart et al. [15]. Finally, we computed intra-individual Spearman correlations of the mRNA abundances determined by the two technologies (RNA-seq and Microarray) and found them to be very strongly correlated (Figure 2C). In Figures 2A through 2C, we have indicated in the labels the ethnic group of each individual – W (White), B (Black) – and placed individuals from the same ethnic group in neighboring rows/columns. As can be seen, the observed high inter-individual mRNA transcript correlations are *independent* of ethnic origin or of the technology used.

### Other categories of transcripts with high inter-individual correlations

**Figure 3:**
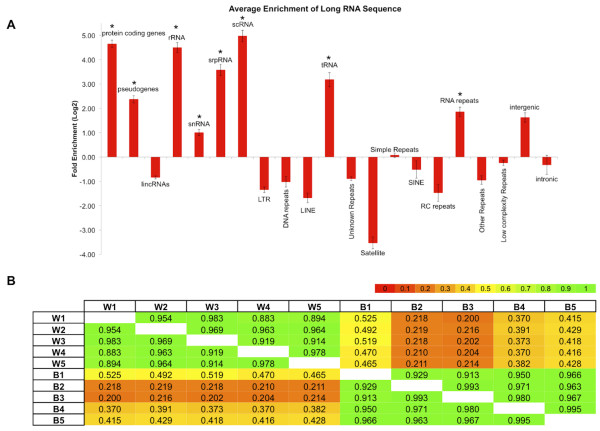
**Enriched elements and pseudogenes. A)** Enrichment analysis of the expressed genomic elements. Shown is the average enrichment for the 10 sequenced samples for various categories of annotated transcripts. The x-axis is the genomic element and the y-axis is the average enrichment value (log2) for each category. Values are averaged across all ten samples. Those categories reaching significant enrichments (P-value < = 0.05) are indicated with a “*”. **B)** Pseudogenes. Heatmap of the inter-individual Pearson correlations of pseudogene transcripts (RNA-seq). The sample IDs are labeled with a W (White) or B (Black).

In view of the very high inter-individual correlations that we observed for the mRNA transcripts, we sought to determine whether other categories of transcripts exhibit similar behavior. To this end, we first examined the various categories of ncRNAs to which reads were mapped uniquely (Figure 1, Table 1) to determine those categories that are enriched in the 10 RNA-seq samples as well as statistically significant. Despite their overall low representation among the sequenced reads (Figure 1A), and in *all* 10 samples, several transcript categories including pseudogenes, rRNA, snRNAs, srpRNAs, tRNAs, scRNAs, and RNA-repeats exhibit statistically significant over-representation compared to a random selection of transcripts (Figure 3A). Subsequent computation of pair-wise Pearson correlations only for transcripts belonging to each of these seven categories also revealed high inter-individual correlation values (Additional file 5), similar to those shown in Figures 2A and 2B. Just as in the case of the protein coding genes, the high-concordance in expression patterns of these categories of ncRNAs, suggest that these transcripts reflect marshaled events.

### Differences in platelet pseudogene transcript expression between the two ethnic groups

As mentioned above, the two racial groups do not show any differences in their mRNA profiles (Figure 2). Neither do they show any differences when comparing their footprints along the rRNA, snRNAs, srpRNAs, tRNAs, scRNAs, and RNA dimensions (Additional file 5). However, when we compared the transcript expression of their pseudogenes (one of the seven statistically significant enriched classes of ncRNAs) we observed a very clear difference between the two groups (Figure 3B). Within each ethnic group the inter-individual *Pearson* correlations were very high; however, across ethnic boundaries there was no correlation. It is important to emphasize that this difference arises when looking at the aggregate expression levels of the pseudogene transcripts as a group rather than specific differentially expressed genes. The use of DEseq [26] reveals only a handful of statistically-significant pseudogenes, including the pseudogenes for mitochondrial genes MTND4P12, MTND1P23 and MTND4P24, and the histone cluster genes HIST1H2BPS2 and HTATSF1P2 (Additional file 6) as well as a few others. As shown in Additional file 1, the only difference we observe between Whites and Blacks is in the amount of hemoglobin. Even though we cannot exclude the possibility of a link between hemoglobin expression and the expression of pseudogenes in platelets such a connection seems unlikely as platelets do not express hemoglobin.

### Correlations between mRNA transcripts and their corresponding proteins

**Figure 4:**
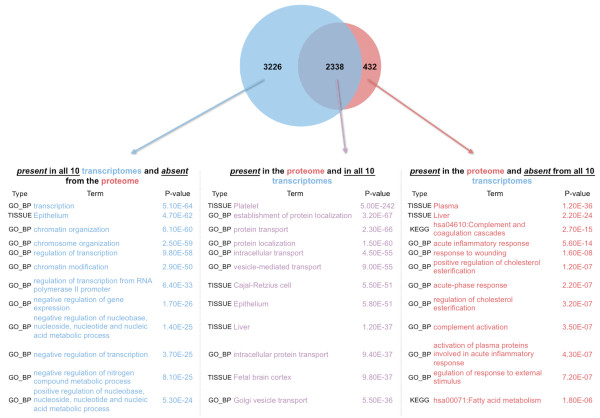
**Three groups of platelet genes. A)** Venn iagram showing the number of genes contained in each of the three shown categories of genes. Note that the five categories are non-overlapping. **B)** Top entries of DAVID analysis for the GO, KEGG pathway, and UP_TISSUE terms corresponding to the genes contained in each of the categories shown in the A) panel.

Having established a high concordance in the composition and abundance of the transcriptome profiles across platelets from different individuals, we sought to characterize the relationship between the platelet transcriptome and the platelet proteome. It is important to note here that such cross-platform comparisons of platelets from different individuals typically present inherent limitations (see Discussion below). We are aware of only a single report that has queried the platelet proteome in a quantitative manner [15] – this is the proteome that we use for our comparisons. From the perspective of the proteome, 3,112 (=3,544-432) or 87.8% of the 3,544 identified proteins have the cognate mRNA present in at least 1 transcriptome, whereas 66% have the cognate mRNA present in *all* 10 transcriptomes (Figure 4A and Additional file 5. The remaining 12.2% (432) of the identified proteins had no counterpart mRNA transcript in any of the 10 RNA-seq datasets. In contrast, of the 10,002 unique mRNAs present in one or more of the 10 RNA-seq transcriptomes, 3,226 (32%) have no corresponding proteome entry. The availability of a quantitative profile for platelet proteins [15] allowed comparison of the *relative abundances* of platelet mRNA transcripts with the relative abundances of platelet proteins (i.e. Spearman rank correlation). We carried out this computation for the 2,338 genes that were represented in both the platelet transcriptome and the platelet proteome and observed a very weak correlation between the two: for RNA-seq the Spearman correlation equaled r = 0.311 (p-value = 2.2E-10) and for microarray data r = 0.312 (p-value = 2.6E-16). See Additional file 7 for the Spearman correlation values of each of the 10 individual transcriptomes. In a separate comparison, if we extend the Spearman computation to the 5,564 mRNAs that are present all 10 transcriptomes and the 3,544 proteins that are present in the proteome, the observed value indicates a poor correlation between the transcriptome and the proteome with r = 0.223 (p-value = 2.1E-11).

### Several identifiable groups of platelet genes and their “Gene Ontology” analysis

We were struck by the substantial number of non-overlapping genes between the transcriptome and proteome, and considered whether this apparent discrepancy might be attributed, in part, to different functional or cellular classes of platelet genes among the various sub-groups. For this analysis, we focused on the following three non-overlapping groups: 1) genes represented by transcripts in all 10 RNA-seq profiles and present in the reference proteome; 2) genes represented by transcripts in all 10 RNA-seq profiles and absent from the reference proteome; and, 3) genes present in the proteome but not in the transcriptome of any of the 10 RNA-seq profiles (Figure 4A). Given that the proteome was reported using UNIPROT identifiers, we first converted the ENSEMBL identifiers of the RNA-seq datasets into UNIPROT: this had the practical consequence of mapping multiple ENSEMBL entries onto a single UNIPROT identifier – in these instances, we paired up the UNIPROT id with the *most abundant* of the ENSEMBL entries. The UNIPROT ids of each group were analyzed using DAVID, and included the Gene Ontology (GO) terms for biophysical processes (GO_BP_FAT), pathway entries (KEGG), and tissue information (UP_TISSUE). Figure 4B lists the top few entries for each group, in order of ascending p-value: for the complete list see Additional file 8. As can be seen, each of the groups of genes in Figure 4 has a distinct profile. We have also extended the analyses and considered two additional gene groups, for a total of five groups: 4) genes represented in the RNA-seq profiles of 1-9 individuals and absent from the proteome, and 5) genes present in the proteome and in the RNA-seq profiles of 1-9 individuals (but not in all 10 individuals). See Additional file 9 for the five gene groups and Additional file 8 for a complete list of the associated terms.

## Discussion

As a first step to understanding the genetic and molecular causes of inter-individual variability in human platelet reactivity, it is important to define the variability in the repertoire of platelet genes. RNA-seq is the most technologically advanced approach to characterizing transcriptomes, but has been applied to human platelets in a rather limited manner. In this report, we present the largest series of human platelet RNA-seq data to date. Using platelets from healthy donors, our major findings are: 1) a very high correlation of both protein-coding transcript composition and abundance among different subjects, a result that is independent of race and of the employed technology; 2) a consensus platelet transcriptome that identifies mRNAs for most biochemically-identified platelet proteins; and, 3) a racial difference among expressed pseudogenes. Furthermore, analysis of the RNA-seq-identified platelet transcriptome that we report and of the mass spectrometry-identified platelet proteins reported by Burkhart et al. revealed the following: 1) most identified proteins had a corresponding mRNA; 2) more than half of the identified platelet mRNAs lacked a corresponding protein; and, 3) the 2,338 genes that are represented in both the platelet transcriptome and the platelet proteome exhibited a very weak but statistically-significant rank correlation. Our data suggests that those mRNAs without a corresponding protein encode proteins with different functions than those that have a corresponding protein in the steady state. These findings, together with the consensus transcriptome from this cohort of healthy males provide an important framework for future patient studies and for new research directions in platelet biology.

Our findings are strengthened by having carefully selected a cohort of 10 males within a narrow age-range and no notable clinical history. Of the 10 individuals, five self-identified as White and five self-identified as Black; in all instances, the self-identification was confirmed independently through genotyping. Moreover, all long RNAs from their platelets’ total RNA were sequenced using a single platform (Life Technologies SOLiD 5500xl). These choices were meant to narrow the range of profile variations and to minimize the impact of potential contaminating events (other cell types, foreign RNA), the latter being stochastic in nature.

Comparisons of the transcript levels obtained with RNA-seq revealed very high *inter*-individual (Pearson) correlations for mRNA (Figure 2), pseudogenes, rRNA, snRNAs, srpRNAs, tRNAs, scRNAs, and RNA repeat elements (Additional file 5). The high correlations of the mRNA profiles were also recapitulated using Affymetrix microarrays, indicating that they are not a function of the employed technology. The concordance of mRNA composition and abundance across 10 different individuals suggests a structured and well-coordinated process. Unexpectedly, although pseudogene expression was highly correlated *within* each of the two racial groups, it was not correlated *across* the groups (Figure 3B). There is little appreciation for racial differences in pseudogene expression, although this has been reported for *DHFRP1*[27]. Pseudogene expression can regulate expression of protein-coding transcripts, and perhaps the differences we observed may contribute to racial differences in platelet function. As we mentioned above, Additional file 1 indicates that the only difference we observe between Whites and Blacks is in the amount of hemoglobin: even though there remains the formal possibility of a link between hemoglobin expression and the expression of pseudogenes in platelets, such a connection seems unlikely as platelets do not express hemoglobin.

There has been a lack of clarity regarding the correlation between the platelet transcriptome and proteome, which was recently highlighted after the first quantitative proteome was reported [11,12,15]. Having produced a reasonable first draft of a reference human platelet transcriptome, we had an opportunity to shed additional light on the relationship between platelet mRNAs and proteins. Although there are limitations (described below) in comparing across platforms that use fundamentally different protocols and chemistries, we were able to confirm that a large portion of the reported platelet proteins (2,338 of 2,770 [83.4%]) had a corresponding reference transcript among those that were common to (i.e. *intersection*) all 10 sequenced individuals. However, we observed a poor correlation in the level of expression among these 2,338 “overlapping” mRNAs/proteins (Spearman rank correlation r = 0.311; p-value = 2.6E-10). Importantly, these genes fell into functional categories of well-established features of platelet physiology (Figure 4, middle list; Additional file 9), such as vesicle trafficking. These analyses provide a high level of confidence that the 2,338 “overlapping” genes (Additional file 8) are authentic to and commonly expressed in human platelets.

We were intrigued by the large number of platelet mRNAs that were present in all 10 studied individuals but for which no corresponding protein was identified (3,226 of 5,564 [57.9%]; Figure 4, left list; Additional file 9; and, Additional file 8). The presence of untranslated RNAs and the exquisitely consistent abundances measured by RNA-seq across the 10 individuals suggest that regulation through mRNA degradation is either limited or controlled, despite the presence of high amounts of platelet miRNAs that has been documented by us [10] and others [14,28]. Potentially, this group of mRNAs could represent mRNAs that: 1) are vestigial megakaryocyte mRNAs with little or no physiologic consequence in peripheral blood platelets; 2) undergo transfer via exosomes or microparticles to other vascular locations (as has been shown for miRNAs [28]); or, 3) will not be translated until they are needed in hemostasis or inflammation. The first possibility may be particularly relevant for those untranslated RNAs present in very low levels in our healthy group of subjects. We expect that the future application of both proteomic and RNA-seq technologies to platelets isolated from the same individuals in both healthy and diseased states will help clarify this picture.

We also found that 16.6% of the reported proteome lacked a corresponding reference mRNA. Analysis of this category of genes (Figure 4, right list; Additional file 8; and, Additional file 9) is most consistent with either platelet endocytosis (e.g. fibrinogen and immunoglobulin) or platelet preparations contaminated with plasma proteins. Finally, we would point out that this emerging picture gets more complicated when considering the inter-individual variation in transcriptomes. Additional file 9 illustrates this point: when we considered transcripts present in only 1-9 of our subjects (but not in all 10), we find an additional 3,634 transcripts that lack a corresponding protein and an additional 774 proteins that lack a corresponding mRNA transcript.

**Figure 5:**
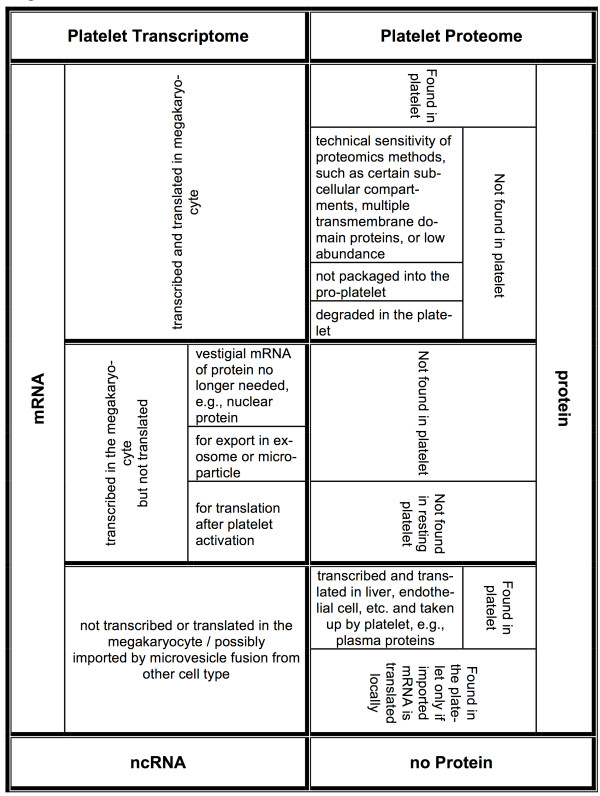
**Relationships between the platelet transcriptome (left) and proteome (right).** The entries comprise some of the known causes that may underlie the observed discordance between platelets mRNAs and platelet proteins

As mentioned above there are limitations to our transcriptome and proteome analyses. The proteome reported by Burkhart et al. was based on a single quantitative mass spectrometry experiment from a pool of 4 platelets [15]. In addition, the reproducibility of proteomic analyses is estimated to be ~65% [15]. Figure 5 describes in more details the relationship between the platelet transcriptome and the platelet proteome. Lastly, the transcriptome-proteome comparisons we carried out unavoidably involved mRNAs and proteins sourced from different individuals. Despite these limitations, it was intriguing to find that the GO term analyses of the different identifiable gene groups correspond to distinct biological categories (Figure 4; Additional file 8), thus providing support for the biologic validity of the relationships we have uncovered. Given the limited overlap that the proteomics findings detailed in the study by Burkhart et al. has with the earlier proteomics reports by Qureshi et al. [9] and by Dowal et al. [22] – detailed in Table S3 of Burkhart et al. – it will be important to focus future efforts on quantitative analyses with technical and biological replicates: it will be particularly illuminating to determine whether the high-inter-individual mRNA correlations carry over to analogous correlations between the platelet proteomes of different individuals.

## Conclusion

Summarily, the very high inter-individual correlations of the transcriptome signatures across 10 different subjects representing two ethnic groups together with the results of our analyses indicate that it is feasible to assemble a platelet mRNA-ome that can serve as a reference for future platelet transcriptomic studies of human health and disease.

## Reviewers comments

### Reviewer #1 (Dr Neil Smalheiser)

I thought this paper was convincing and well written. It provides a good systems biology contribution to platelet biology. I only have one minor comment/question. On p. 9, you mention certain Spearman correlations in the 0.3 range as modest and significant, yet later in the same paragraph you have another correlation listed as r = 0.223 as "no correlation" even though the p-value shows extremely high significance. Please clarify and revise what you mean.

Response: *We thank the Reviewer for pointing this out – it has been corrected in the final version.*

### Reviewer #2 (Dr Mikhail Dozmorov - nominated by Dr Yuri Gusev)

The manuscript by Londin et al. addresses an important topic of investigating human platelets transcriptome among individuals and ethnicities. Moreover, the authors performed correlation of the transcriptome with publicly available proteome dataset, and report several interesting observations. The manuscript is very well written, clear and concise in each and every part. All potential questions that come up during reading the manuscript are answered either later in the text, or in supplementary material. The methods are flawless, and also original, as the authors describe their technique of not just annotating genes in the transcriptome, but also consider repeat elements, non-coding regions, and distinguish between intronic and intergenic regions. All data are prepared to be made available upon publication.

The manuscript is recommended for publication without revisions.

### Reviewer #3 (Dr Eugene Koonin)

This is a very interesting, very clearly written paper that demonstrates the robustness of the up to date RNAseq protocols and reveals remarkable features of the platelet transcriptome and proteome. Probably, the most important observation reported here is the very strong inter-individual correlation between the transcriptomes. This finding lends confidence to other observations. Among these, it is notable that the union of the transcripts detected in platelets from 10 individuals accounts to about half of the entire set of human protein-coding genes which is an unexpectedly large number. It is of further interest that over half of these transcripts are untranslated or at best weakly translated. This work clearly provides a platform for probing the biology of platelets and a template for analogous studies on other cell and tissue types.

minor comments not for publication:

When comparing the black and white cohorts, the authors speak of "ethnic groups" under Results and "races" under Discussion. On this sensitive issue, it is advisable to use a uniform and most broadly accepted terminology, whatever that is.

Response: *We have changed all instances of “races” to “ethnic groups” throughout the manuscript.*

“(≤ 1/10,000 the of β-actin)” isn't this supposed to be >1/10,000 the of β-actin

Response: *This should have been “≥1/10,000” and has been corrected.*

## Competing interests

The authors declare that they have no competing interests.

## Authors’ contributions

Contributions: IR, ERL, PFB, and SEM designed the study. PFB, SEM, LE and CS recruited the study subjects; ERL, IR, EH, PL, analyzed the data; PF and KD performed the next-generation sequencing; IR, ERL, PFB, and SEM contributed to writing and reviewing the manuscript; All authors approved of the manuscript.

## Supplementary Material

Additional file 1 Subject demographics. Click here for file

Additional file 2 **Genetic ancestry of study participants.** Shown are the principal components of the genetic ancestry of study participants derived from genotype data. Click here for file

Additional file 3 Summary of sequencing mapping. Click here for file

Additional file 4 **Genes observed to be expressed within 13 PCR cycles of ACTB and observed in all 10 subjects.** Shown are all the genes that were expressed within 13 PCR cycles of ACTB (≥ 1/10,000 of *β*-actin’s expression) in all ten of the individuals studied. The ENSEMBL gene ID along with the common gene name are given. Click here for file

Additional file 5 **Pearson correlations of non-protein coding transcripts.** Click here for file

Additional file 6 Differentially expressed pseudogenes. Click here for file

Additional file 7 **Transcriptome and proteome correlations.(A)** A schematic representation of Venn diagrams of the potential overlaps of genes present in the transcriptome, proteome or both. **(B)** Individual Spearman correlations of the genes expressed in the transcriptome and proteome (purple groupings from the Venn diagrams in **A**), or the genes expressed in transcriptome or proteome (all expressed genes). Correlations were performed using transcriptome data derived from either RNA-seq or from a microarray. Click here for file

Additional File 8 GO terms for the five identifiable groups of protein coding transcripts. Click here for file

Additional file 9 **Five identifiable groups of protein-coding transcripts.** Shown are the overlaps of five groupings of protein-coding transcripts, based upon the total number of samples observed in the transcriptome or proteome. Click here for file
