# Supplementary material for: The human platelet: strong transcriptome correlations among individuals associate weakly with the platelet proteome
Source: Biol Direct. 2014 Feb 14;9:3. doi: 10.1186/1745-6150-9-3 (PMC3937023; doi:10.1186/1745-6150-9-3)
Supplement: Additional file 1 — Subject demographics. [file 1745-6150-9-3-S1.docx]

Additional File 1. A) Subject Demographics. B) Subject Demographics by race.

A.

| **Characteristic** | **Value** |
| --- | --- |
| Age (mean +/- SD) | 28.1 +/-7.63 |
| Males (n) | 10 |
| Self-identified race |  |
| White | 5 |
| Black | 5 |
| Current Smokers | 3 |
| Hypertension (n) | 0 |
| BMI (mean +/- SD) | 25.1 +/- 2.87 |
| Hemoglobin (g/dl) (mean +/- SD) | 13 +/- 0.83 |
| Platelet Count (x10^5^/μl) (mean +/- SD) | 198 +/- 51.2 |

B.

| **Characteristic** | **Value Black (n=5)** | **Value White (n=5)** | ***P-*value** |
| --- | --- | --- | --- |
| Age (mean +/– SD) | 29.6 +/– 10.2 | 26.6 +/– 5.94 | 0.59 |
| Sex (% Male) | 100% | 100% |  |
| Smoker % | 2/5 (40%) | 1/5 (20%) |  |
| BMI (mean +/– SD) | 25.0 +/– 3.66 | 25.3 +/– 2.67 | 0.89 |
| Hemoglobin (g/dl) (mean +/- SD) | 12.4 +/– 0.94 | 13.6 +/– 0.24 | 0.03 |
| Platelet Count (x 10^5^/μl) (mean +/– SD) | 206 +/– 32.8 | 189 +/– 75.8 | 0.63 |
