# Supplementary figures and images for: The human platelet: strong transcriptome correlations among individuals associate weakly with the platelet proteome
Source: Biol Direct. 2014 Feb 14;9:3. doi: 10.1186/1745-6150-9-3 (PMC3937023; doi:10.1186/1745-6150-9-3)

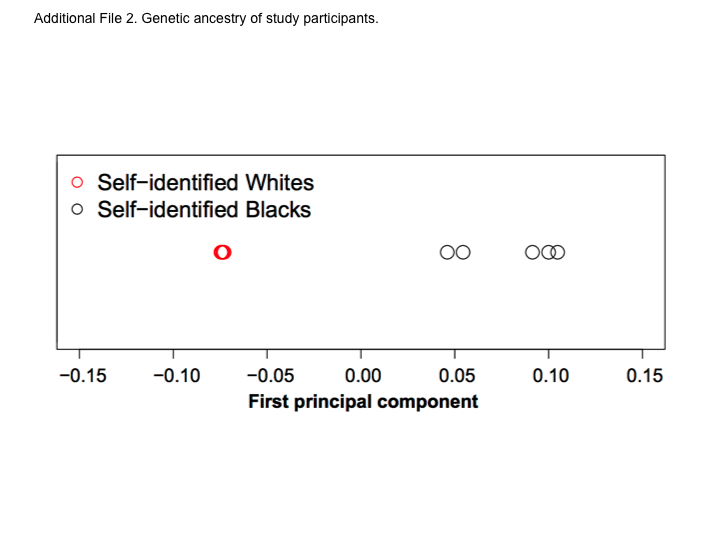

Supplement: Additional file 2 — Genetic ancestry of study participants. Shown are the principal components of the genetic ancestry of study participants derived from genotype data. [file 1745-6150-9-3-S2.tiff]

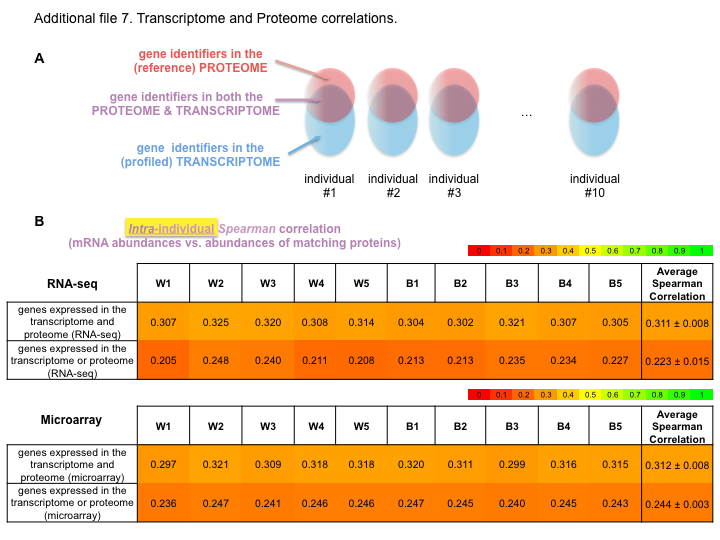

Supplement: Additional file 7 — Transcriptome and proteome correlations.(A) A schematic representation of Venn diagrams of the potential overlaps of genes present in the transcriptome, proteome or both. (B) Individual Spearman correlations of the genes expressed in the transcriptome and proteome (purple groupings from the Venn diagrams in A), or the genes expressed in transcriptome or proteome (all expressed genes). Correlations were performed using transcriptome data derived from either RNA-seq or from a microarray. [file 1745-6150-9-3-S7.tiff]

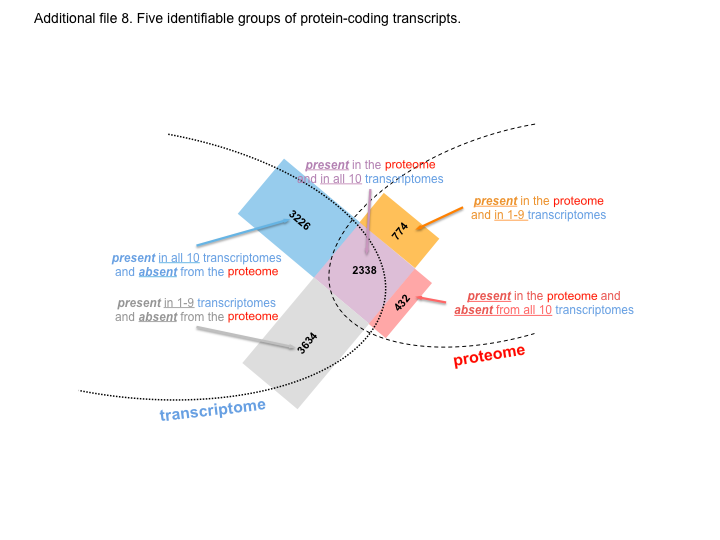

Supplement: Additional file 9 — Five identifiable groups of protein-coding transcripts. Shown are the overlaps of five groupings of protein-coding transcripts, based upon the total number of samples observed in the transcriptome or proteome. [file 1745-6150-9-3-S9.tiff]
