# Supplementary material for: The human platelet: strong transcriptome correlations among individuals associate weakly with the platelet proteome
Source: Biol Direct. 2014 Feb 14;9:3. doi: 10.1186/1745-6150-9-3 (PMC3937023; doi:10.1186/1745-6150-9-3)
Supplement: Additional file 3 — Summary of sequencing mapping. [file 1745-6150-9-3-S3.docx]

Additional file 3. Summary of sequence mapping.

| **Subject** | **Total Starting Reads** | **Total Uniquely Mapped Reads** | **Percent Mapped Uniquely** |
| --- | --- | --- | --- |
| W1 | 162,594,999 | 61,951,917 | 38.10% |
| W2 | 155,555,072 | 64,627,541 | 41.55% |
| W3 | 159,471,944 | 68,281,402 | 42.82% |
| W4 | 161,023,328 | 66,351,905 | 41.21% |
| W5 | 155,412,342 | 61,804,073 | 39.77% |
| B1 | 157,628,599 | 60,485,074 | 38.37% |
| B2 | 148,659,147 | 60,213,554 | 40.50% |
| B3 | 162,800,750 | 73,337,350 | 45.05% |
| B4 | 155,835,847 | 65,740,060 | 42.19% |
| B5 | 165,379,619 | 66,133,616 | 39.99% |
|  |  |  |  |
| **Average** | **158,436,165** | **64,892,649** | **40.96%** |
