# Supplementary material for: The human platelet: strong transcriptome correlations among individuals associate weakly with the platelet proteome
Source: Biol Direct. 2014 Feb 14;9:3. doi: 10.1186/1745-6150-9-3 (PMC3937023; doi:10.1186/1745-6150-9-3)
Supplement: Additional file 6 — Differentially expressed pseudogenes. [file 1745-6150-9-3-S6.docx]

| Pseudogene | log 2 Fold Change | pval |
| --- | --- | --- |
| ENSG00000225972_ MTND1P23 | 7.8171 | 6.95E-09 |
| ENSG00000244398_ RP11-466H18.1 | 2.386 | 0.032 |
| ENSG00000232495_ AC069287.1 | -2.118 | 0.032 |
| ENSG00000177359_ RP11-551L14.1 | -inf | 0.004 |
| ENSG00000178550_ AC010170.1 | -1.120 | 0.005 |
| ENSG00000124399_ RP11-663P9.2 | -3.125 | 0.029 |
| ENSG00000247627_ MTND4P12 | 3.632 | 3.08E-09 |
| ENSG00000271361_ HTATSF1P2 | -3.681 | 0.0022 |
| ENSG00000217646_ HIST1H2BPS2 | 3.418 | 0.028 |
| ENSG00000234025_ RP11-257K9.3 | Inf | 0.0243 |
| ENSG00000232177_ MTND4P24 | 4.841 | 1.05E-11 |

Additional File 6. Differentially expressed pseudogenes.
